# Supplementary material for: The accuracy of absolute differential abundance analysis from relative count data
Source: PLoS Comput Biol. 2022 Jul 11;18(7):e1010284. doi: 10.1371/journal.pcbi.1010284 (PMC9302745; doi:10.1371/journal.pcbi.1010284)
Supplement: S1 Text — This file contains supplemental methods and results detailing the characteristics of simulated data sets, describes variable importance determination in predictive models, and gives further details on the effects of mitigation strategies like control genes. (PDF) [file pcbi.1010284.s001.pdf]

Supplemental information for *The accuracy of absolute differential abundance analysis from relative count data*

Kimberly E. Roche[1*], Sayan Mukherjee[123]

1. Program in Computational Biology and Bioinformatics, Duke University, Durham, NC 27708, United States
2. Departments of Statistical Science, Mathematics, Computer Science, Biostatistics & Bioinformatics, Duke University, Durham, NC 27708, United States; Institute for Computer Science, Universität Leipzig and the Max Planck Institute for Mathematics in the Natural Sciences, Leipzig, 04103, Germany
3. Center for Genomic and Computational Biology, Duke University, Durham, NC 27708, United States

Characteristics of the simulated data

The properties of simulated data sets in terms of percent differentially abundant features, change in abundance across conditions, and percent zero counts are shown in S1 Fig. Most simulations featured a minority of differentially abundant features and the distribution of the proportion of differentially abundant features was similar across feature number settings (100, 1000, and 5000 features). Fold change in total abundance between conditions was similar across settings as well. The percent zero counts in our simulations increased as the number of features increased. This is because, on average, simulations with a larger number of features had larger overall absolute total abundances and were more likely to be downsampled when "observed" relative abundances were drawn, yielding dropouts.
Differential abundance was simulated as a random per-feature perturbation applied to a subset of randomly selected features. As such, net change in abundance across the composition was generally asymmetric. S2 Fig illustrates the distribution of differentially abundant features experiencing an *increase* in abundance from simulated conditions A to B.

Visualizing outcomes from simulated data

We present alternative summaries of outcomes for all 5625 simulated data sets in this Supplement. Sensitivities and specificities organized by number of simulated features and labeled for percent of differentially abundant features are given in S3 Fig and summaries of these outcomes are available in S1 Table. Alternatively, we present per-setting results - equivalent to those presented in main text Fig 1 - but labeled for simulated fold change in S4 Fig.

Variable importance in predictive models

All features utilized in predictive models are listed in S2 Table. Further, in S3 and S4 Tables, we show the top several most important features (as scored by gain in accuracy upon feature inclusion) for each of the six predictive models over sensitivity and specificity for each of ALDEx2, ANCOM-BC, DESeq2, edgeR (with TMM), and scran.
In all models over sensitivity, features summarizing the prevalence of low-count elements in the composition were highly informative. For all methods except scran, an estimates of the correlation of centered logratio features - summarizing shared change relative to the mean - were also highly informative.
Specificity was best predicted by two sets of features: 1) those describing the percent of features apparently undergoing substantial change across simulated conditions and 2) the scale of that change, as estimated by the standard deviation of observed feature-level change across conditions.

Results in real data

Per-method sensitivity and specificity outcomes in real data are summarized in S5 and S6 Tables respectively. Counts of false positivies as a function of percent differentially abundant features are given in S6 Fig. See main text Fig 2 for the analogous results from simulated data.

Increased stringency improves agreement between absolute and relative calls

In the main text we tested for per-feature differential abundance using p-values derived from each of the methods explored after multiple test correction (via Benjamini-Hochberg) at a significance level of $\alpha \leq 0.05$. False positive counts for these results are given in main text Fig 2.

Here, we introduce a higher bar for differential abundance: a differentially abundant feature must be statistically significant with FDR ≤ 0.01 *and* to exhibit a fold change of at least 2 (i.e. either a doubling or halving) on average across conditions to be considered truly differentially abundant. We evaluated the agreement between calls made in absolute and relative data using these criteria, in essence, asking about the degree to which data types agree of the most unambiguous differences. These results are summarized in S7 and S8 Figs. Median false positive counts for each of ANCOM-BC, DESeq2, and edgeR (with TMM) were decreased on average by 112, 101, and 102 respectively. For scran, this average FP count decrease was 54.7. For ALDEx2 were calls essentially unchanged (average change in median FP count = 0.667).

Specificity improvement in DESeq2 using control genes

Most of the methods we evaluated rescale the data against some per-sample measure of central tendency. An alternative to this procedure is to rescale observed abundances against an explicit reference quantity - for example, against spike-in abundances, as we have done to construct ”absolute” abundances from published data sets. DESeq2's ”control_genes” feature allows a user to specify a set of putatively stable reference features against which observed abundances can be rescaled before testing for differential abundance. We applied this control_genes feature in three ways: 1) using known stable features in simulated data, 2) using known stable features in real data, and 3) using a set of ”housekeeping” genes derived from the literature [1, 2, 3, 4].

First, for each of the 5625 simulated data sets, we selected 10 features from a given data set which were at or below the 10th percentile in terms of coefficient of variation in absolute counts. These are some of the stablest features in the ”true” abundances. In the corresponding relative abundance data, we identified these as control genes using the argument of the same name in DESeq2 and performed differential abundance accuracy analysis as described in the main text. The median false positive rate (FPR) across all simulated data sets was reduced from 0.124 to 0.083 and in simulated data sets with a majority of differentially abundant features, median FPR was reduced by more than 50%, from 0.385 to 0.197. Distributions of these outcomes are shown in S9 Fig where gains in sensitivity are minimal but gains in specificity are generally substantial and positive.

In real data too, DESeq2 sensitivity and specificity were generally improved by the use of ”low-variance” reference features as controls, though these improvements were often modest. These results are given in S7 and S8 Tables.

Finally, we utilized thirteen ”housekeeping” genes derived from four published studies [1, 2, 3, 4]: *atp5pb*, *b2m*, *cycs*, *eer1a1*, *eef2*, *gapdh*, *hmbs*, *hprt1*, *lmna*, *ppib*, *prkg1*, *tbcb*, and *tbp*. We utilized these features as controls in six of the real data sets utilizing human or mouse expression data. These results are given in S2 and S3 Tables. Sensitivity and specificity were improved in the data sets of Song et al. and Hashimshony et al., remained largely unchanged in Muraro et al. and Hagai et al., and actually worsened in Grüen et al. and Monaco et al. This result emphasizes the need for caution in the selection of housekeeping genes

as reference features, as these genes' products may be expected to exhibit variation across tissue types, organisms, and experimental settings [5].

References

1. de Kok JB, Roelofs RW, Giesendorf BA, Pennings JL, Waas ET, Feuth T, et al. Normalization of gene expression measurements in tumor tissues: comparison of 13 endogenous control genes. Lab Invest. 2005;85(1):154–159.

2. Padovan-Merhar O, Nair GP, Biaesch AG, Mayer A, Scarfone S, Foley SW, et al. Single mammalian cells compensate for differences in cellular volume and DNA copy number through independent global transcriptional mechanisms. Mol Cell. 2015;58(2):339–352.

3. Panina Y, Germond A, Masui S, Watanabe TM. Validation of Common Housekeeping Genes as Reference for qPCR Gene Expression Analysis During iPS Reprogramming Process. Sci Rep. 2018;8(1):8716.

4. Nazet U, Schröder A, Grässel S, Muschter D, Proff P, Kirschneck C. Housekeeping gene validation for RT-qPCR studies on synovial fibroblasts derived from healthy and osteoarthritic patients with focus on mechanical loading. PLoS One. 2019;14(12):e0225790.

5. Thorrez L, Laudadio I, Van Deun K, Quintens R, Hendrickx N, Granvik M, et al. Tissue-specific disallowance of housekeeping genes: the other face of cell differentiation. Genome Res. 2011;21(1):95–105.
