# Supplementary material for: The accuracy of absolute differential abundance analysis from relative count data
Source: PLoS Comput Biol. 2022 Jul 11;18(7):e1010284. doi: 10.1371/journal.pcbi.1010284 (PMC9302745; doi:10.1371/journal.pcbi.1010284)
Supplement: S1 Table — Specificity in simulated data, FDR ≤ 0.05. Per-method results are grouped and sorted by increasing feature number. (PDF) [file pcbi.1010284.s002.pdf]

**S1 Table:** Specificity in simulated data, FDR $\leq 0.05$. Per-method results are grouped and sorted by increasing feature number.

| Feature number | Method | Median specificity | Percent of data sets below 95% specificity | Percent of data sets below 50% specificity |
|---|---|---|---|---|
| 100 | ALDEx2 | 0.857 | 64% | 26% |
| 100 | ANCOM-BC | 0.904 | 72% | 12% |
| 100 | DESeq2 | 0.841 | 82% | 16% |
| 100 | edgeR (TMM) | 0.903 | 71% | 15% |
| 100 | scran | 0.885 | 75% | 12% |
| 1000 | ALDEx2 | 0.925 | 58% | 9% |
| 1000 | ANCOM-BC | 0.859 | 87% | 12% |
| 1000 | DESeq2 | 0.853 | 79% | 14% |
| 1000 | edgeR (TMM) | 0.869 | 84% | 16% |
| 1000 | scran | 0.903 | 71% | 8% |
| 5000 | ALDEx2 | 0.942 | 54% | 2% |
| 5000 | ANCOM-BC | 0.822 | 87% | 14% |
| 5000 | DESeq2 | 0.879 | 73% | 9% |
| 5000 | edgeR (TMM) | 0.86 | 83% | 12% |
| 5000 | scran | 0.925 | 63% | 4% |
