# Supplementary material for: The accuracy of absolute differential abundance analysis from relative count data
Source: PLoS Comput Biol. 2022 Jul 11;18(7):e1010284. doi: 10.1371/journal.pcbi.1010284 (PMC9302745; doi:10.1371/journal.pcbi.1010284)
Supplement: S2 Table — Features of observed abundances used to predict sensitivity and specificity outcomes. (PDF) [file pcbi.1010284.s003.pdf]

**S2 Table:** Features of observed abundances used to predict sensitivity and specificity outcomes.

| Feature symbol | Description |
|---|---|
| P | feature number |
| PARTIAL | totals expected to be partially informative (e.g. spike-in normalized) |
| TOTALS_C_FC | absolute fold change in mean totals (A vs. B) |
| TOTALS_C_D | absolute change in mean totals |
| TOTALS_C_MAX_D | max delta in totals |
| TOTALS_C_MED_D | median delta in totals |
| TOTALS_C_SD_D | std. dev. of in totals |
| CORR_RA_MED | median correlation of relative abundances |
| CORR_RA_SD | std. dev. of correlation of relative abundances |
| CORR_RA_SKEW | skew of correlation of relative abundances |
| CORR_LOG_MED | median correlation of log counts |
| CORR_LOG_SD | std. dev. of correlation of log counts |
| CORR_LOG_SKEW | skew of correlation of log counts |
| CORR_CLR_MED | median correlation of CLR features |
| CORR_CLR_SD | std. dev. of correlation of CLR features |
| CORR_CLR_SKEW | skew of correlation of CLR features |
| COMP_C_P0_A | percent features = 0 in condition A |
| COMP_C_P0_B | percent features = 0 in condition B |
| COMP_C_P1_A | percent features = 1 in condition A |
| COMP_C_P1_B | percent features = 1 in condition B |
| COMP_C_P5_A | percent features $\leq 5$ in condition A |
| COMP_C_P5_B | percent features $\leq 5$ in condition B |
| COMP_RA_P01_A | percent features $< 0.1\%$ relative abundance in condition A |
| COMP_RA_P01_B | percent features $< 0.1\%$ relative abundance in condition B |
| COMP_RA_P1_A | percent features $< 1\%$ relative abundance in condition A |
| COMP_RA_P1_B | percent features $< 1\%$ relative abundance in condition B |
| COMP_RA_P5_A | percent features $< 5\%$ relative abundance in condition A |
| COMP_RA_P5_B | percent features $< 5\%$ relative abundance in condition B |
| COMP_RA_MAX_A | max relative abundance in condition A |
| COMP_RA_MED_A | median relative abundance in condition A |
| COMP_RA_SD_A | std. dev. of relative abundance in condition A |
| COMP_RA_SKEW_A | skew of relative abundance in condition A |
| COMP_RA_MAX_B | max relative abundance in condition B |

| | |
|---|---|
| COMP_RA_MED_B | median relative abundance in condition B |
| COMP_RA_SD_B | std. dev. of relative abundance in condition B |
| COMP_RA_SKEW_B | skew of relative abundance in condition B |
| COMP_C_ENT_A | entropy in condition A |
| COMP_C_ENT_B | entropy in condition B |
| FW_RA_MAX_D | max change in relative abundance |
| FW_RA_MED_D | median change in relative abundance |
| FW_RA_SD_D | std. dev. of change in relative abundance |
| FW_RA_PPOS_D | percent features with + change in relative abundances |
| FW_RA_PNEG_D | percent features with - change in relative abundances |
| FW_RA_PFC05_D | percent features with < 0.5 FC in relative abundance |
| FW_RA_PFC1_D | percent features with < 1 FC in relative abundance |
| FW_RA_PFC2_D | percent features with < 2 FC in relative abundance |
| FW_LOG_MAX_D | max change in log counts |
| FW_LOG_MED_D | median change in log counts |
| FW_LOG_SD_D | std. dev. of change in log counts |
| FW_LOG_PPOS_D | percent features with + change in log counts |
| FW_LOG_PNEG_D | percent features with - change in log counts |
| FW_LOG_PFC05_D | percent features with < 0.5 FC in log counts |
| FW_LOG_PFC1_D | percent features with < 1 FC in log counts |
| FW_LOG_PFC2_D | percent features with < 2 FC in log counts |
| FW_CLR_MAX_D | max change in CLR |
| FW_CLR_MED_D | median change in CLR |
| FW_CLR_SD_D | std. dev. of change in CLR |
| FW_CLR_PPOS_D | percent features with + change in CLR |
| FW_CLR_PNEG_D | percent features with - change in CLR |
| FW_CLR_PFC05_D | percent features with < 0.5 FC in CLR |
| FW_CLR_PFC1_D | percent features with < 1 FC in CLR |
| FW_CLR_PFC2_D | percent features with < 2 FC in CLR |
