# Supplementary material for: The accuracy of absolute differential abundance analysis from relative count data
Source: PLoS Comput Biol. 2022 Jul 11;18(7):e1010284. doi: 10.1371/journal.pcbi.1010284 (PMC9302745; doi:10.1371/journal.pcbi.1010284)
Supplement: S3 Table — Predictive features and their relative importance (as gain) in the prediction of sensitivity. (PDF) [file pcbi.1010284.s004.pdf]

S3 Table: Predictive features and their relative importance (as gain) in the prediction of sensitivity.

Method	Feature	Importance
ALDEx2	percent features = 0 in condition B	1
ALDEx2	skew of correlation of CLR features	0.78
ALDEx2	median correlation of CLR features	0.51
ANCOM-BC	percent features = 1 in condition A	1
ANCOM-BC	percent features $\leq$ 5 in condition A	0.82
ANCOM-BC	median correlation of CLR features	0.79
DESeq2	percent features $\leq$ 5 in condition B	1
DESeq2	median correlation of CLR features	0.94
DESeq2	percent features = 1 in condition B	0.74
edgeR (TMM)	median correlation of CLR features	1
edgeR (TMM)	skew of correlation of CLR features	0.74
edgeR (TMM)	percent features = 0 in condition B	0.71
scran	percent features $\leq$ 5 in condition B	1
scran	percent features = 1 in condition A	0.95
scran	percent features $\leq$ 5 in condition A	0.94
