# Supplementary material for: The accuracy of absolute differential abundance analysis from relative count data
Source: PLoS Comput Biol. 2022 Jul 11;18(7):e1010284. doi: 10.1371/journal.pcbi.1010284 (PMC9302745; doi:10.1371/journal.pcbi.1010284)
Supplement: S4 Table — Predictive features and their relative importance (as gain) in the prediction of specificity. (PDF) [file pcbi.1010284.s005.pdf]

S4 Table: Predictive features and their relative importance (as gain) in the prediction of specificity.

Method	Feature	Importance
ALDEx2	percent features with < 0.5 FC in CLR	1
ALDEx2	std. dev. of change in log counts	0.45
ALDEx2	percent features $= 0$ in condition B	0.39
ANCOM-BC	percent features with < 0.5 FC in CLR	1
ANCOM-BC	percent features with < 0.5 FC in log counts	0.39
ANCOM-BC	std. dev. of change in log counts	0.37
DESeq2	percent features with < 0.5 FC in CLR	1
DESeq2	std. dev. of change in log counts	0.44
DESeq2	percent features with < 2 FC in CLR	0.42
edgeR (TMM)	percent features with < 0.5 FC in CLR	1
edgeR (TMM)	percent features with < 2 FC in CLR	0.44
edgeR (TMM)	std. dev. of change in log counts	0.36
scran	percent features with < 0.5 FC in CLR	1
scran	std. dev. of change in log counts	0.51
scran	percent features with < 2 FC in CLR	0.34
