# Supplementary material for: The accuracy of absolute differential abundance analysis from relative count data
Source: PLoS Comput Biol. 2022 Jul 11;18(7):e1010284. doi: 10.1371/journal.pcbi.1010284 (PMC9302745; doi:10.1371/journal.pcbi.1010284)
Supplement: S6 Table — Observed specificities on real data sets. (PDF) [file pcbi.1010284.s007.pdf]

S6 Table: Observed specificities on real data sets.

Data set	ALDEx2	ANCOM-BC	DESeq2	edgeR (TMM)	scran
Hagai et al. [1]	0.983	0.825	0.968	0.96	0.862
Hashimshony et al. [2]	1	0.987	0.972	0.967	0.99
Song et al. [3]	0.771	0.624	0.653	0.719	0.865
Monaco et al. [4]	0.991	0.634	0.83	0.77	1
Vieira-Silva et al. [5]	0.975	0.75	0.95	0.875	0.925
Barlow et al. [6]	1	0.927	0.909	0.855	1
Gruen et al. [7]	0.913	0.671	0.921	0.878	0.794
Muraro et al. [8]	0.97	0.778	0.853	0.904	0.834
Kimmerling et al. [9]	1	0.994	0.998	0.993	0.993
Yu et al. [10]	0.773	0.56	0.855	0.754	0.536
Owens et al. [11]	0.147	0.064	0.082	0.04	0.147
Klein et al. [12]	0.036	0.018	0.089	0.018	0.018

References

1. Hagai T, Chen X, Miragaia RJ, Rostom R, Gomes T, Kunowska N, et al. Gene expression variability across cells and species shapes innate immunity. Nature. 2018;563(7730):197–202.

2. Hashimshony T, Senderovich N, Avital G, Klochendler A, de Leeuw Y, Anavy L, et al. CEL-Seq2: sensitive highly-multiplexed single-cell RNA-Seq. Genome Biol. 2016;17:77.

3. Song SG, Kim S, Koh J, Yim J, Han B, Kim YA, et al. Comparative analysis of the tumor immune-microenvironment of primary and brain metastases of non-small-cell lung cancer reveals organ-specific and EGFR mutation-dependent unique immune landscape. Cancer Immunol Immunother. 2021;70(7):2035–2048.

4. Monaco G, Lee B, Xu W, Mustafah S, Hwang YY, Carré C, et al. RNA-Seq Signatures Normalized by mRNA Abundance Allow Absolute Deconvolution of Human Immune Cell Types. Cell Rep. 2019;26(6):1627–1640.e7.

5. Vieira-Silva S, Sabino J, Valles-Colomer M, Falony G, Kathagen G, Caenepeel C, et al. Quantitative microbiome profiling disentangles inflammation- and bile duct obstruction-associated microbiota alterations across PSC/IBD diagnoses. Nat Microbiol. 2019;4(11):1826–1831.

6. Barlow JT, Bogatyrev SR, Ismagilov RF. A quantitative sequencing framework for absolute abundance measurements of mucosal and lumenal microbial communities. Nat Commun. 2020;11(1):2590.

7. Grün D, Kester L, van Oudenaarden A. Validation of noise models for single-cell transcriptomics. Nat Methods. 2014;11(6):637–640.

8. Muraro MJ, Dharmadhikari G, Grün D, Groen N, Dielen T, Jansen E, et al. A Single-Cell Transcriptome Atlas of the Human Pancreas. Cell Syst. 2016;3(4):385–394.e3.

9. Kimmerling RJ, Prakadan SM, Gupta AJ, Calistri NL, Stevens MM, Olcum S, et al. Linking single-cell measurements of mass, growth rate, and gene expression. Genome Biol. 2018;19(1):207.

10. Yu Y, Fuscoe JC, Zhao C, Guo C, Jia M, Qing T, et al. A rat RNA-Seq transcriptomic BodyMap across 11 organs and 4 developmental stages. Nat Commun. 2014;5(1):1–11.

11. Owens NDL, Blitz IL, Lane MA, Patrushev I, Overton JD, Gilchrist MJ, et al. Measuring Absolute RNA Copy Numbers at High Temporal Resolution Reveals Transcriptome Kinetics in Development. Cell Rep. 2016;14(3):632–647.

12. Klein AM, Mazutis L, Akartuna I, Tallapragada N, Veres A, Li V, et al. Droplet Barcoding for Single-Cell Transcriptomics Applied to Embryonic Stem Cells. Cell. 2015;161(5):1187–1201.
