# Supplementary material for: The accuracy of absolute differential abundance analysis from relative count data
Source: PLoS Comput Biol. 2022 Jul 11;18(7):e1010284. doi: 10.1371/journal.pcbi.1010284 (PMC9302745; doi:10.1371/journal.pcbi.1010284)
Supplement: S7 Table — DESeq2 sensitivity when using a variety of features as controls (via the control_genes argument). Sensitivity is improved when using a random set of low-variance features as references against which to rescale observed abundances. A selection of “housekeeping” genes used for the same purpose gives less improvement over baseline. (PDF) [file pcbi.1010284.s008.pdf]

S7 Table: DESeq2 sensitivity when using a variety of features as controls (via the control_genes argument). Sensitivity is improved when using a random set of low-variance features as references against which to rescale observed abundances. A selection of "housekeeping" genes used for the same purpose gives less improvement over baseline.

Data set	No controls	Low-variance features	Housekeeping genes
Song et al. [1]	0.472	0.947	0.866
Monaco et al. [2]	0.373	0.454	0.432
Muraro et al. [3]	0.316	0.391	0.315
Hagai et al. [4]	0.465	0.5	0.476
Hashimshony et al. [5]	0.104	0.201	0.253
Gruen et al. [6]	0.748	0.785	0.447

References

1. Song SG, Kim S, Koh J, Yim J, Han B, Kim YA, et al. Comparative analysis of the tumor immune-microenvironment of primary and brain metastases of non-small-cell lung cancer reveals organ-specific and EGFR mutation-dependent unique immune landscape. Cancer Immunol Immunother. 2021;70(7):2035–2048.

2. Monaco G, Lee B, Xu W, Mustafah S, Hwang YY, Carré C, et al. RNA-Seq Signatures Normalized by mRNA Abundance Allow Absolute Deconvolution of Human Immune Cell Types. Cell Rep. 2019;26(6):1627–1640.e7.

3. Muraro MJ, Dharmadhikari G, Grün D, Groen N, Dielen T, Jansen E, et al. A Single-Cell Transcriptome Atlas of the Human Pancreas. Cell Syst. 2016;3(4):385–394.e3.

4. Hagai T, Chen X, Miragaia RJ, Rostom R, Gomes T, Kunowska N, et al. Gene expression variability across cells and species shapes innate immunity. Nature. 2018;563(7730):197–202.

5. Hashimshony T, Senderovich N, Avital G, Klochendler A, de Leeuw Y, Anavy L, et al. CEL-Seq2: sensitive highly-multiplexed single-cell RNA-Seq. Genome Biol. 2016;17:77.

6. Grün D, Kester L, van Oudenaarden A. Validation of noise models for single-cell transcriptomics. Nat Methods. 2014;11(6):637–640.
