# Supplementary material for: The accuracy of absolute differential abundance analysis from relative count data
Source: PLoS Comput Biol. 2022 Jul 11;18(7):e1010284. doi: 10.1371/journal.pcbi.1010284 (PMC9302745; doi:10.1371/journal.pcbi.1010284)
Supplement: S8 Table — DESeq2 specificity when using a variety of features as controls (via the control_genes argument). Specificity is improved when using a random set of low-variance features as references against which to rescale observed abundances. A selection of “housekeeping” genes used for the same purpose gives less improvement or worsens specificity in the data sets of Monaco et al. and Grüen et al. (PDF) [file pcbi.1010284.s009.pdf]

S8 Table: DESeq2 specificity when using a variety of features as controls (via the control_genes argument). Specificity is improved when using a random set of low-variance features as references against which to rescale observed abundances. A selection of "housekeeping" genes used for the same purpose gives less improvement or worsens specificity in the data sets of Monaco et al. and Grüen et al.

Data set	No controls	Low-variance features	Housekeeping genes
Song et al. [1]	0.653	0.902	0.886
Monaco et al. [2]	0.83	0.896	0.575
Muraro et al. [3]	0.853	0.906	0.872
Hagai et al. [4]	0.968	0.975	0.97
Hashimshony et al. [5]	0.972	0.986	0.989
Gruen et al. [6]	0.921	0.924	0.748

References

1. Song SG, Kim S, Koh J, Yim J, Han B, Kim YA, et al. Comparative analysis of the tumor immune-microenvironment of primary and brain metastases of non-small-cell lung cancer reveals organ-specific and EGFR mutation-dependent unique immune landscape. Cancer Immunol Immunother. 2021;70(7):2035–2048.

2. Monaco G, Lee B, Xu W, Mustafah S, Hwang YY, Carré C, et al. RNA-Seq Signatures Normalized by mRNA Abundance Allow Absolute Deconvolution of Human Immune Cell Types. Cell Rep. 2019;26(6):1627–1640.e7.

3. Muraro MJ, Dharmadhikari G, Grün D, Groen N, Dielen T, Jansen E, et al. A Single-Cell Transcriptome Atlas of the Human Pancreas. Cell Syst. 2016;3(4):385–394.e3.

4. Hagai T, Chen X, Miragaia RJ, Rostom R, Gomes T, Kunowska N, et al. Gene expression variability across cells and species shapes innate immunity. Nature. 2018;563(7730):197–202.

5. Hashimshony T, Senderovich N, Avital G, Klochendler A, de Leeuw Y, Anavy L, et al. CEL-Seq2: sensitive highly-multiplexed single-cell RNA-Seq. Genome Biol. 2016;17:77.

6. Grün D, Kester L, van Oudenaarden A. Validation of noise models for single-cell transcriptomics. Nat Methods. 2014;11(6):637–640.
