# Supplementary figures and images for: The accuracy of absolute differential abundance analysis from relative count data
Source: PLoS Comput Biol. 2022 Jul 11;18(7):e1010284. doi: 10.1371/journal.pcbi.1010284 (PMC9302745; doi:10.1371/journal.pcbi.1010284)

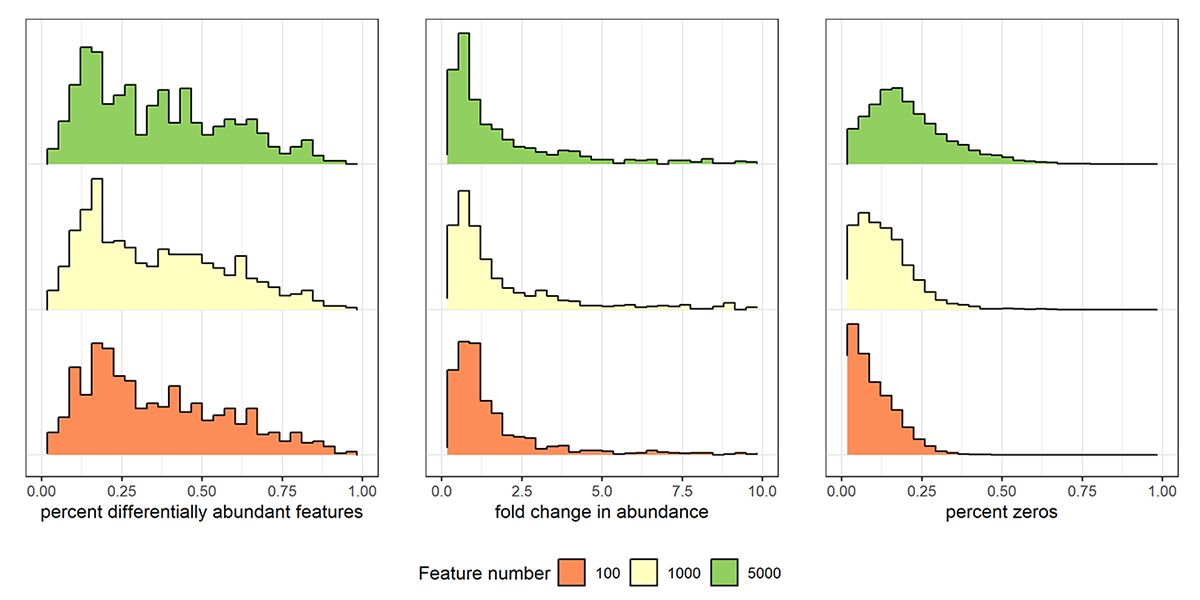

Supplement: S1 Fig — Distributions associated with three characteristics of the 5625 simulated data sets: a) percent differentially abundant features, b) fold change in total abundance across conditions, and c) percent zeros. (TIF) [file pcbi.1010284.s010.tif]

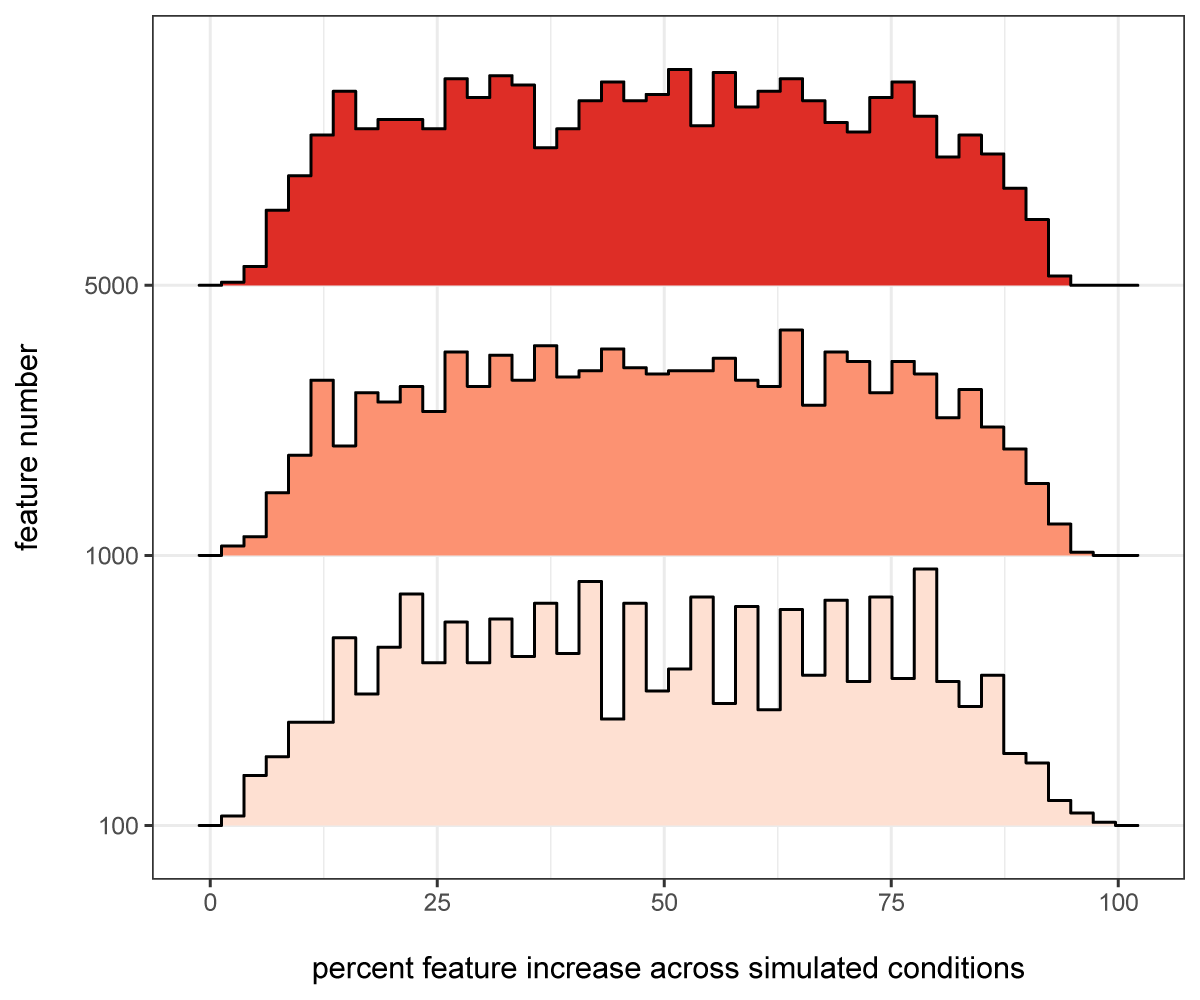

Supplement: S2 Fig — The number, scale, and direction of differential abundance varied randomly in simulation, giving rise to both increases and decreases in total abundance across conditions. (TIF) [file pcbi.1010284.s011.tif]

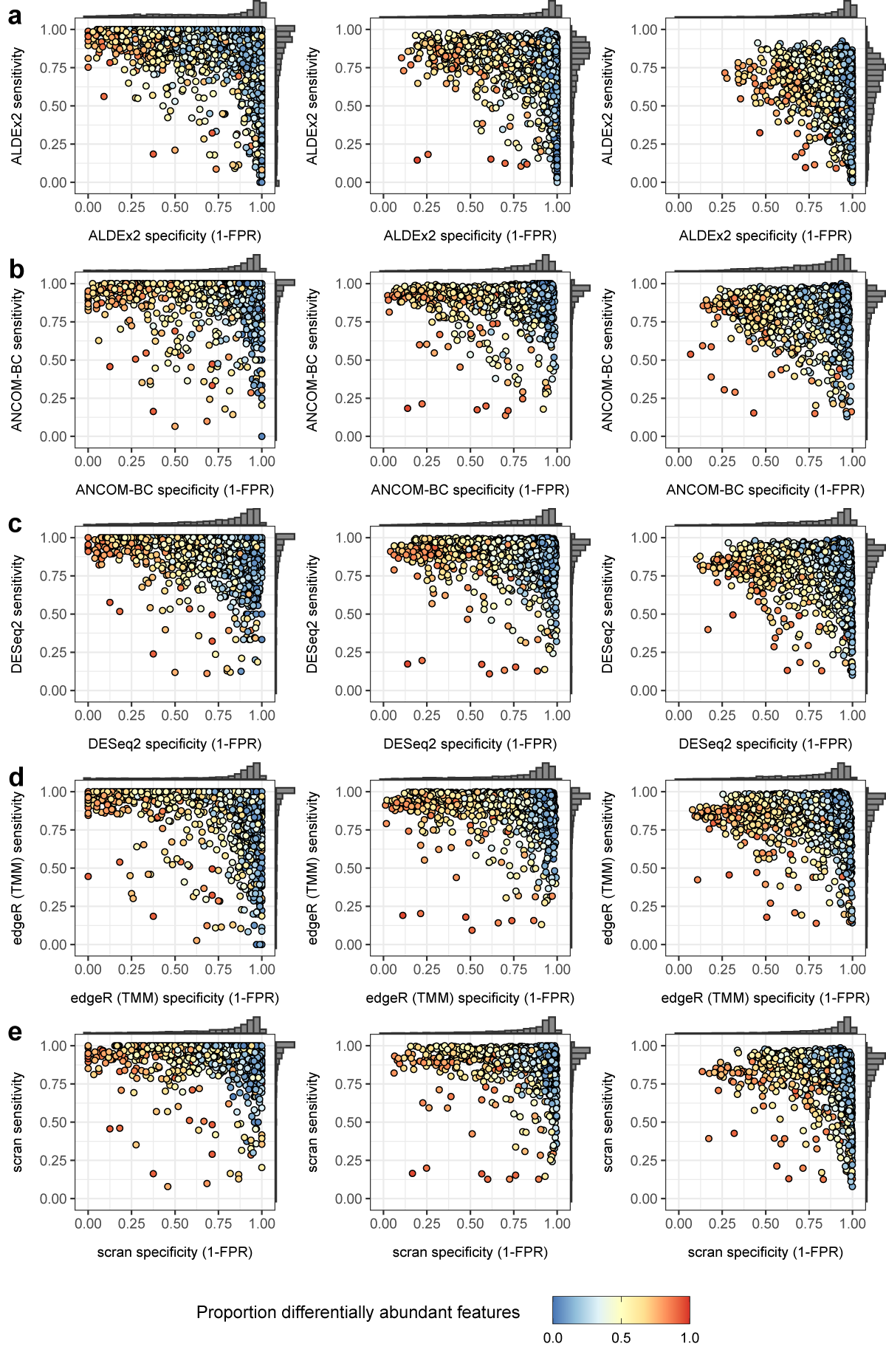

Supplement: S3 Fig — Sensitivity and specificity for five differential abundance calling methods in terms of increasing feature number from a) 100 to b) 1000 to c) 5000 features. (TIF) [file pcbi.1010284.s012.tif]

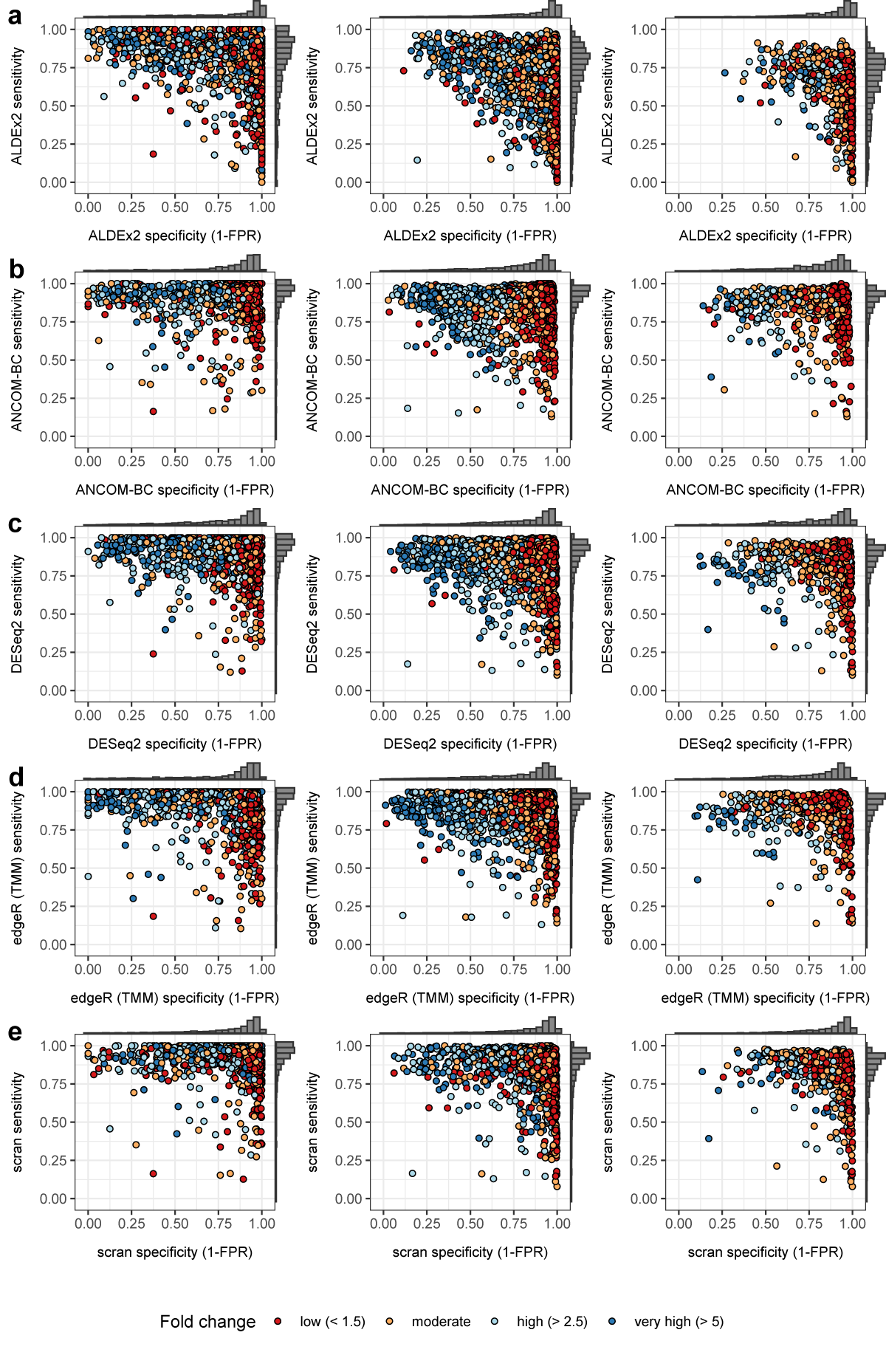

Supplement: S4 Fig — Sensitivity and specificity for five differential abundance calling methods in three experimental settings: a) Microbial, b) Bulk Transcriptomic, and c) Cell Transcriptomic settings. Data sets are labeled by fold change across conditions. (TIF) [file pcbi.1010284.s013.tif]

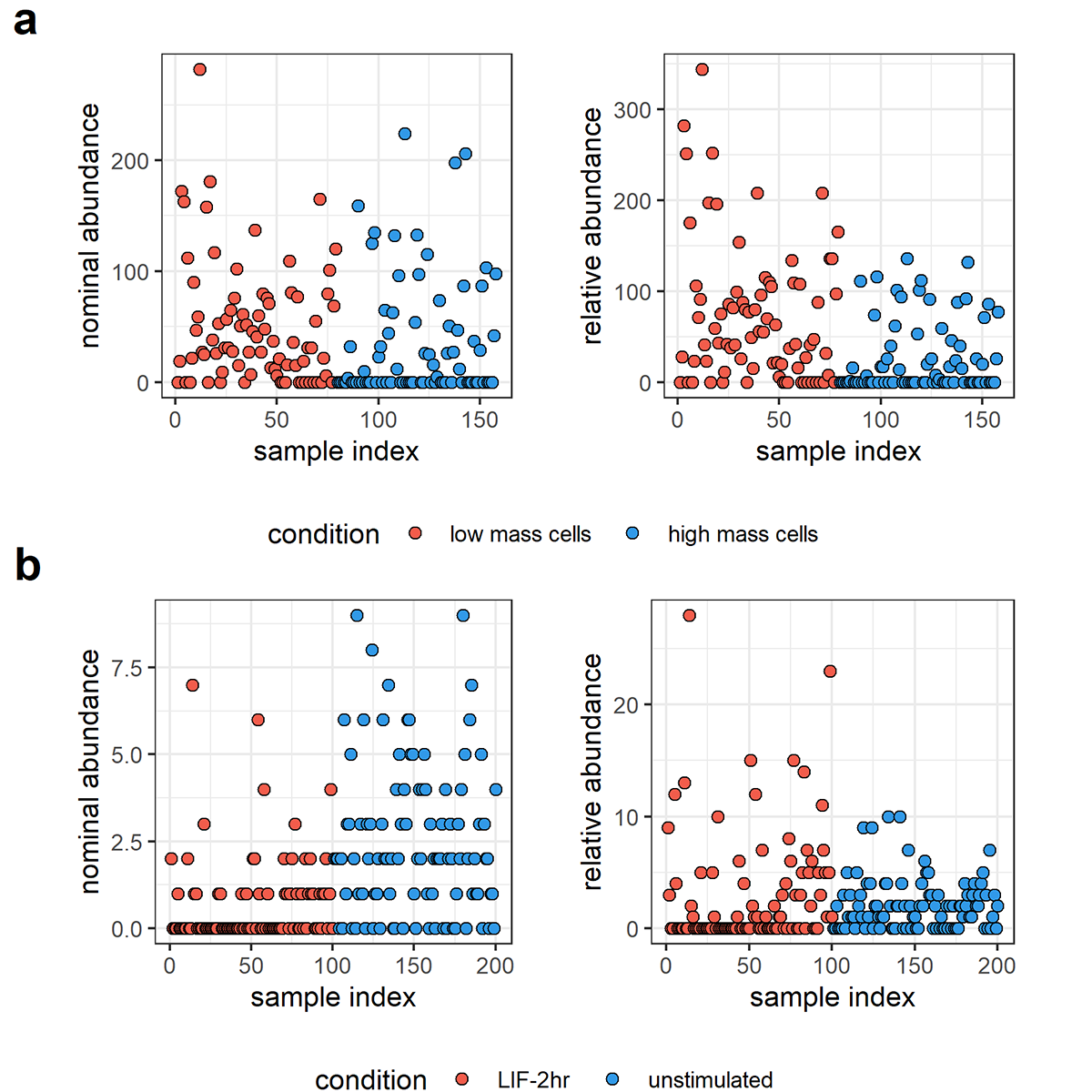

Supplement: S5 Fig — Discrepant calls in nominal and relative abundances. a) A typical false negative result in data derived from the experiment of Kimmerling et al. This feature (the gene H2-T3) is significantly differentially abundant in the nominal abundances but not in estimates made from relative abundances by DESeq2. b) A typical false positive result in data from Klein et al. associated with gene UNG, tested by edgeR (with TMM normalization). (TIF) [file pcbi.1010284.s014.tif]

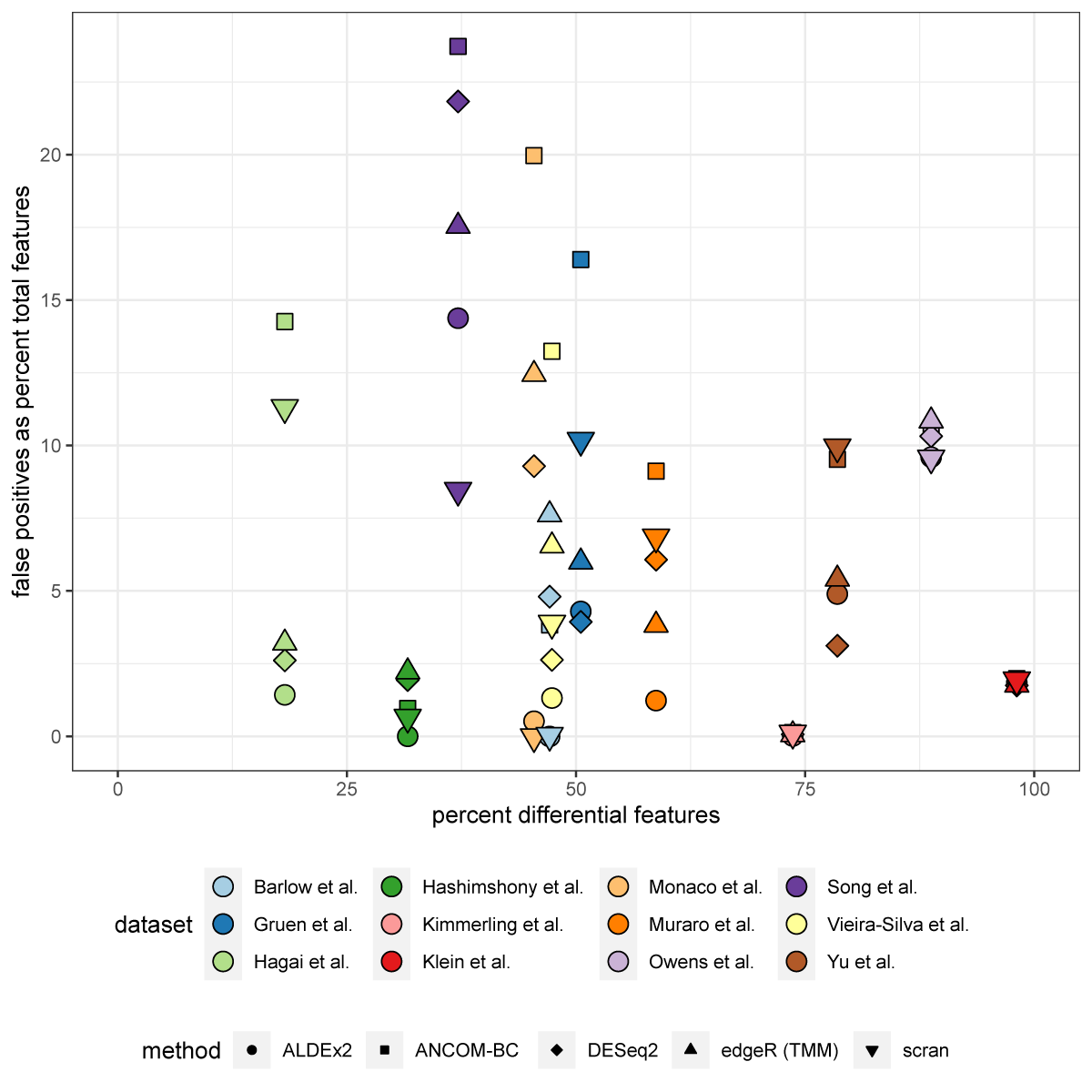

Supplement: S6 Fig — Counts of false positives as a function of the percent of simulated differentially abundant features for five methods applied to 12 real data sets. (TIF) [file pcbi.1010284.s015.tif]

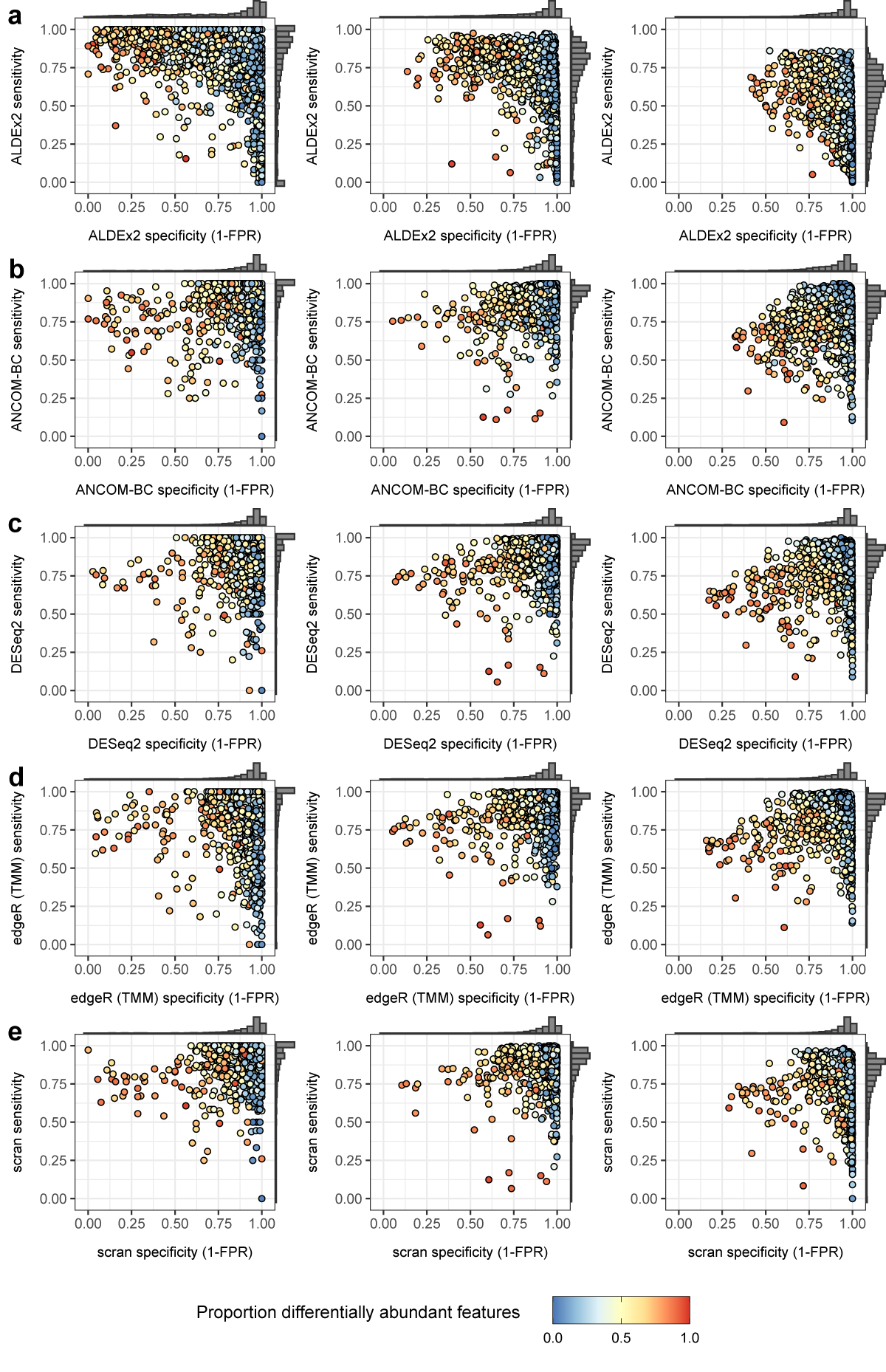

Supplement: S7 Fig — Sensitivity and specificity for five differential abundance calling methods in three experimental settings: a) Microbial, b) Bulk Transcriptomic, and c) Cell Transcriptomic settings. Data sets are labeled by proportion of differentially abundant features. Here, differential abundance calling is subject to greater stringency: FDR ≤ 0.01 and a fold change across conditions of at least 2. Median specificity is improved from 0.90 (FDR ≤ 0.05) to 0.95. (TIF) [file pcbi.1010284.s016.tif]

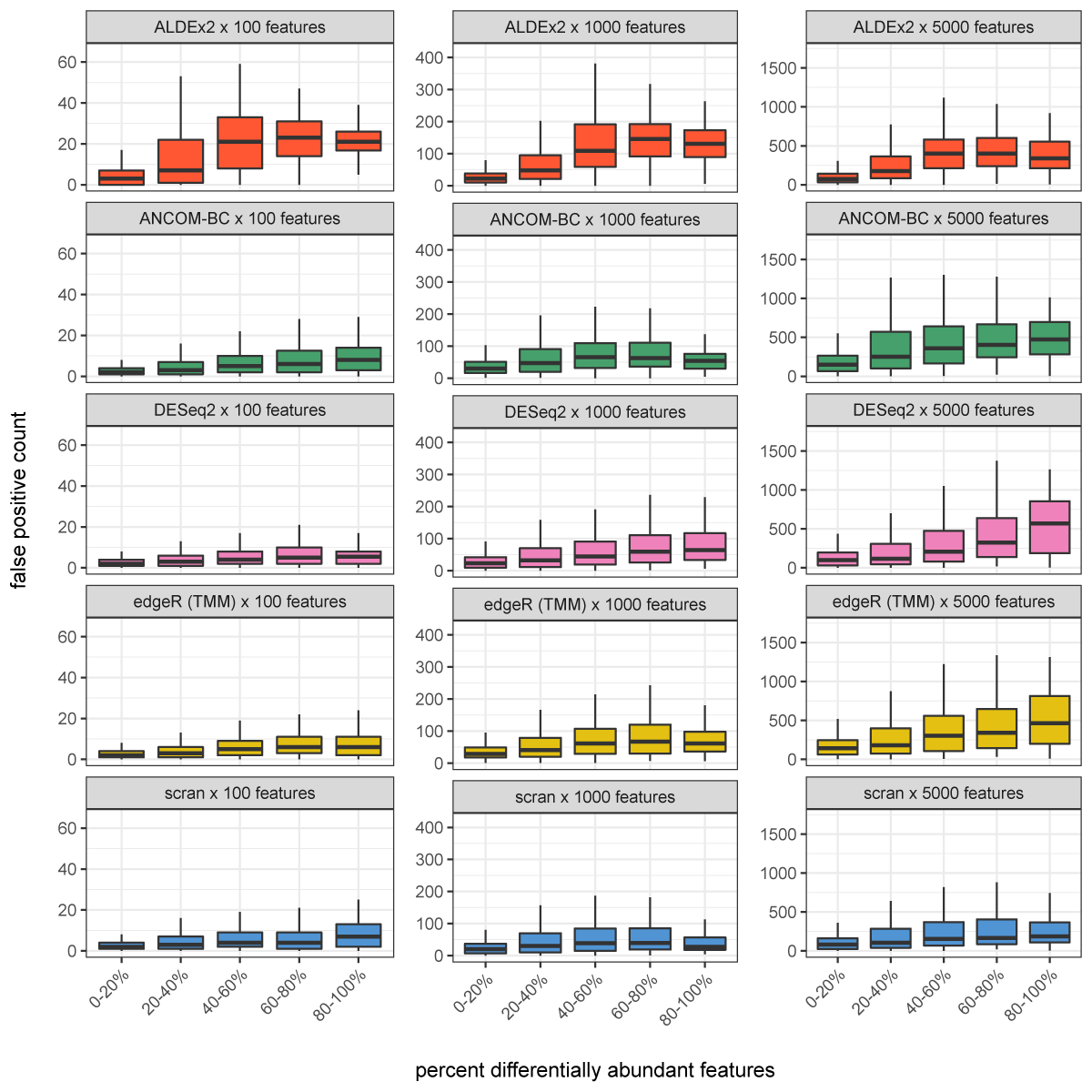

Supplement: S8 Fig — Counts of false positives as a function of the percent of simulated differentially abundant features for five methods with stringent differential abundance calling (FDR ≤ 0.01 and a fold change across conditions of at least 2). Columns segregate simulations with increasing numbers of features. Counts of false positives are reduced relative to FDR ≤ 0.05. (TIF) [file pcbi.1010284.s017.tif]

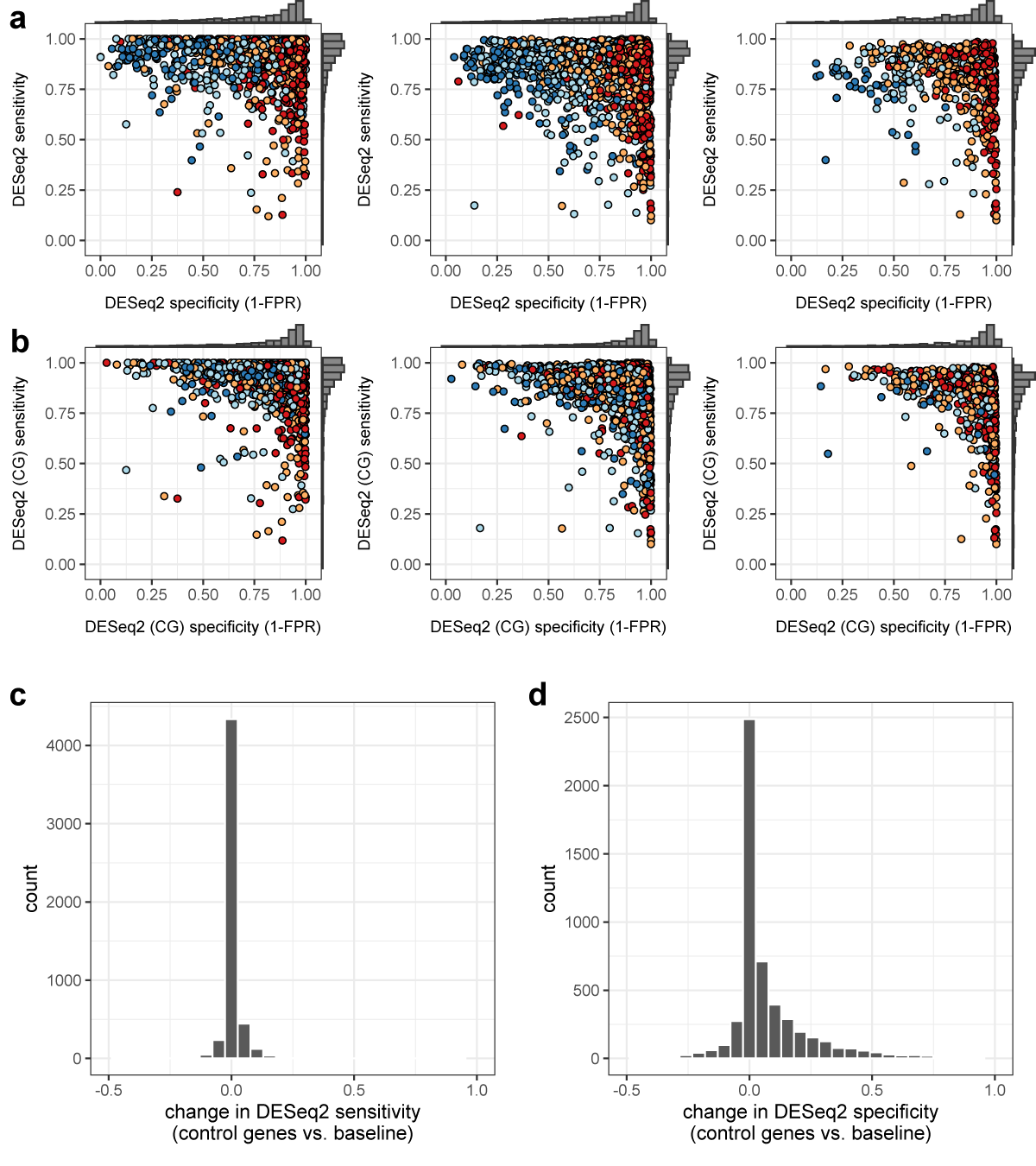

Supplement: S9 Fig — Sensitivity and specificity for all simulated data sets using DESeq2 a) without and b) with “control genes.” Per-columns settings from left to right are Microbial, Transcriptomic (center column), and Bulk Transcriptomic. c) Distribution of the change in sensitivity and d) specificity following the introduction of control genes. Sensitivity is largely unchanged. Specificity is generally improved. (TIF) [file pcbi.1010284.s018.tif]
