# Supplementary material for: The accuracy of absolute differential abundance analysis from relative count data
Source: PLoS Comput Biol. 2022 Jul 11;18(7):e1010284. doi: 10.1371/journal.pcbi.1010284 (PMC9302745; doi:10.1371/journal.pcbi.1010284)
Supplement: Attachment — Submitted filename: responses_to_reviewers.docx [file pcbi.1010284.s019.docx]

May 11, 2022

Dear Dr. Li,

Thank you for consideration of this manuscript and for sharing the comments of both reviewers. We have considered these comments and made a number of changes to the manuscript which we think improve the presentation of results greatly. We thank the reviewers and editors for their thoughtful perspective and hope the changes detailed below will address the issues identified.

Responses to individual reviewer comments are highlighted in blue below and line numbers, where referenced, refer to locations in the updated manuscript. We have attached updated manuscripts both with and without highlights (in blue) indicating changes and we hope this new version is suitable for publication in PLOS Computational Biology. Thank you again for your time and consideration.

Sincerely,

Kimberly E. Roche

Email: [kimberly.e.roche@gmail.com](mailto:kimberly.e.roche@gmail.com)

Phone: +1 864 376-5754

Following comments from Reviewer #1 about a lack of diversity in the methods tested, we have incorporated both edgeR and ANCOM-BC into existing analyses for a wider perspective. edgeR has been widely used for differential expression testing in RNA-seq data for almost two decades. ANCOM-BC is an additional method originating explicitly from 16S metagenomics work. We show the performance of these additional methods is comparable to others in Figures 1 and 2.

Also, in incorporating edgeR, it was necessary to make changes to the processing of “observed” counts upstream of differential abundance testing. Previously, observed total abundances in both simulated and real data sets were normalized to give relative count data in the form of **counts per million reads**. This normalization step has been removed and results in all tables and figures have been updated to reflect this, generally small, change.

Reviewer #1: This paper describes an investigation into the use of relative abundance measurements and normalization to infer absolute abundance. This would be of interest to several different fields of researchers all using sequencing data. It's really nice to see that this has been applied across sequencing applications covering RNA sequencing and 16S sequencing. The paper is well written and easy to follow, however I have a few recommendations, mostly minor editing.

We thank the reviewer for the positive outlook on the value of this work.

Recommendations
Box 1 is really informative, but should be referred to within the main body of text.

Excellent point. This reference has been added the Introduction on line 49.

Line 127 Supplemental figures remain as question marks. Line 192 Supplemental table remains as a question mark.

Our apologies for this oversight. These and other broken references have been fixed.

Inconsistent use of capital letters for "transcriptomic setting" and "microbial setting" (see lines 148-152 for example).

We have updated this such that Transcriptomic and Microbial are capitalized any place in the text these words are used to reference simulated “settings” designed to mimic these data types.

I think Line 209 should be referring to figure 3 and figure 4? Otherwise figure 3 is not referred to. Figure 7 is referred to in the text before figure 6.

Figure and table numbering in the main text has been corrected so that all references should be correct and appear in the order in which those elements are referenced in the text.

On the work with the real data, these have been split into three sets of studies when describing the results. The criteria for this split is unclear and should be explictly described.

This is a great point. We have revised the Real data portion of the Results section heavily and we now divide these real data sets into three broad categories based of observed outcomes: 1) low sensitivity and high specificity, 2) intermediate sensitivity and specificity, and 3) high sensitivity, low specificity. This rationale is described (only as briefly as I have done here) starting on line 259.

There is a description of the differences between the normalization methods employed by DESeq2, scran and ALDEx2 in the methods but I think this needs to be moved to the introduction, alongside a stronger justification of the choice of software.

This is a great suggestion. We have moved the introduction of methods to the end of the Introduction section itself and more clearly state our rationale for their choice: each of these methods performs some kind of heuristic rescaling of the observed total counts, often based on some measure of central tendency within a sample. In particular, we expected these methods to be more accurate than others which simply normalize for total abundance or library size. In support of this last point, we incorporate the accuracy estimates of differential abundance testing using edgeR with only library size normalization in Figure 2. Counts of false positives are much higher for this method than in other, rescaling-based methods.

edgeR is arguably used a lot more often for RNA sequencing studies - why was this not considered?

This is a great point. We attempted to select a minimal set of methods which utilized some kind of rescaling strategy but because edgeR is so widely utilized – and other readers are likely to expect it to be included – we have added results from edgeR to existing figures and tables. We note that its performance is comparable to other methods where TMM normalization has been used. See Figures 1 and 2.

scran has been designed for single cell data - is it appropriate to be using this for bulk RNA and 16S data? Justification of this is needed. It feels like you're actually primarily picking single cell analysis software and then using these with bulk RNA seq and 16s data - are you actually interested in single cell analysis or all three analysis methods?

While it is true that scran was developed for single-cell sequence count data, we are not aware of anything in its methodology that would preclude its use on sequence count data more generally – e.g. key assumptions about sparsity or data format. We have included scran because of its unique stepwise normalization scheme, which has been widely adopted enough to appear in a recent benchmarking study in single-cell (e.g. Luecken et al. 2021). As at least one other study has found that single-cell and bulk RNA-seq methods achieve comparable performance on 16S metagenomics data as compared to methods “designed” for that data (Calgaro et al. 2020), we believe that in this case, to the extent our “abstract” simulated count data are a reasonable stand-in for real experimental data, each of these methodologies represented by ALDEx2, ANCOM-BC, DESeq2, edgeR and scran are general purpose enough to be directly compared here.

I think you should be mentioning that none of the software selected are designed for use with 16S data - all have been developed with RNA sequencing data in mind. Therefore it's not surprising that the microbiome data performs poorly. This could also provide an opportunity to discuss the adoption of these softwares in the 16S research community and whether it is suitable.

ALDEx2 was chosen for inclusion because of its increasing use in 16S metagenomics studies where “compositional data analysis” methods have been advocated. The reviewer is correct however, that we have failed to include any 16S-exclusive methods in our study. To that end, we have added results from ANCOM-BC to all existing analyses. ANCOM-BC was developed for 16S metagenomics data and utilizes its own rescaling strategy for observed total counts. Again however, we believe its model is general purpose enough that it should be broadly applicable to relative count data of the types we study here.

Typo line 364 - "txhese" should be "these"

This has been fixed.

Where 6000 simulations has been mentioned in the text, this shoud be changed to the actual number used - 5625.

We have updated the text to reference the exact 5625 everywhere this total is mentioned.

The methods describe the statistics underlying each piece of software but no details are given on what cut off has been employed for each software for describing significantly differentially abundant e.g. FDR < 0.05. This should be stated.

We apologize for this omission. All differential abundance “calls” were made using corrections for FDR < 0.05. We have added additional results which utilize more stringent significance criteria as well to show that accuracy – in terms of agreement between differential abundance calls on absolute and relative count data – is improved by a combination of more stringent significance and effect size thresholds. See the “Mitigation strategies” section starting on line 310.

Figure 7 - no description of what red and blue means?

We apologize for this omission. Those labels, indicating differential experimental conditions, have been added to the figure.

From the paper, you're suggesting that spike ins are needed for more accurate abundance calculations but you've under stated this in the discussion. More emphasis should be given to this point.

We have taken the reviewer’s suggestion and stressed the advantages of control features (like spike-ins) in the Discussion, starting on line 349. These comments are bolstered by an additional analysis – suggested by Reviewer #2 and added to the main text (starting line 320) and Supplement of this manuscript which employs the “control_genes” feature of DESeq2. In short, when a small set of 10-20 “stable” features is available against which total per-sample abundances can be normalized, both sensitivity and specificity are increased.

Reviewer #2: The authors present a large scale assessment of the sensitivity and specificity of statistical methods that attempt to detect differential features from count datasets, without being provided with information about changes in the true total abundance across samples. While previous papers have taken more unequivocal positions on this question, e.g. that certain classes of methods must be used and others can never be successfully applied to experiments producing matrices of counts, this work I think rightly considers how the violation of model assumptions may result in loss of accuracy in degree, and furthermore shows that to some extent the loss of accuracy can be predicted by dataset features. This can hopefully guide practitioners to choosing methods that are likely to be reliable given particular types and features of their data. For example, DESeq2 and ALDEx2 had a relatively high number (15-18%) of simulated datasets with very low specificity (<50%) for the simulation of microbiome data, while this is much less likely for simulated bulk RNA-seq data (1-2%). While different simulation settings will capture data attributes of different data sets, any particular one simulation setting doesn't tell a complete story. The important contribution from this work can be seen in Figures 1 and 2, where by sweeping across a broad range of simulation settings, one can easily see that proportion of differential features or extent of effect sizes results in loss of specificity across methods.
We should bring to the reviewer’s attention that updates to sample processing meant to accommodate edgeR have altered these results such that the difference in performance between “Microbial” and “Bulk Transcriptomic” settings is less pronounced for DESeq2 in particular, which suffers from a slightly larger proportion of exceptionally high false positive cases.

Major comments:

It was hard from the simulation model description to get a sense for the scale of fold changes in the various simulations. It would help to include a histogram or plot showing the quantiles of the true LFC distribution for various example simulated datasets. In particular the tails.
We agree. The distributions of differentially abundant features and fold change in total abundance in simulated data sets has been included as Supplemental Figure 1 and referenced in the main text where the simulation procedure is first described (line 150).

Were the simulated fold changes always symmetric? It seemed this way from the equation for theta^(2). I would recommend to have at least a few simulations of asymmetric fold changes as these should lead to even quicker degradation of performance for methods that are not provided with information about the true abundance scale, as they often target the middle of the distribution of observed sample-to-sample ratios.
The simulated data sets were *not* symmetric with respect to the direction or magnitude of differentially abundant features. Specifically per-feature perturbations were drawn at random and applied to a subset of randomly selected features. The resulting change in total abundance across features was generally not symmetric and resulted in net decreases in abundance about as often as net increases. The distribution of percent of features experiencing an increasing in abundance from simulated conditions A to B is given in Supplemental Figure 2 and coupled with Supplemental Figure 1, we hope this sketches a more complete picture of the range of simulated change.

I didn't see what was the threshold used for assessing a positive call (5%, 10%?).
Reviewer #1 noted this omission as well and we apologize for the oversight. All differential abundance “calls” were made using corrections for FDR < 0.05. We have also added additional results in the main text in a “Mitigation strategies” section (starting line 310) and in the Supplement which utilize stringent significance criteria to show that accuracy – in terms of agreement between differential abundance calls on absolute and relative count data – is improved by a combination of more stringent significance and effect size thresholds. That is, it is much more likely for absolute and relative abundances to agree on the “big differences.”

DESeq2 since it was originally released has the option to provide informative features ("controlGenes") in order to estimate the true abundance scale per sample (in the estimateSizeFactors function, the first step in DE analysis). It may be useful to know in this context how a small amount of information may or may not help methods that accept this type of information (maybe other methods can as well). In simulations in which a set of stable features exist, how do results change if 10 or 20 of such stable features are provided to the methods for the purpose of scaling factor estimation? For the real data examples, what if housekeeping genes (Gapdh, etc.) are provided to methods that accept these stable reference features? Do results change qualitatively?
This is an excellent suggestion and we have added a brief additional analysis related to the use of these control features with DESeq2 and improvements in the accuracy of differential abundance calling which derive from their use. In short, using a set of “stable” features via the control_genes argument of DESeq2, we saw an across-the-board increase in both sensitivity and specificity of differential abundance calls from relative count data as compared to calls made on absolute counts. For example, in data sets with a majority of differentially abundant features – arguably the most challenging cases – median false positive rates were reduced by more than 50% from 0.385 to 0.197.

We also attempted to use a set of housekeeping genes pulled from the literature on six of the data sets presented in the “Real data” section which were derived from human or mouse studies. These genes included *gapdh, hprt1*, and eleven others, purportedly stable under certain experimental conditions. Results on accuracy outcomes varied here, with sensitivity and specificity improving or remaining largely unchanged in 4 of 6 data sets when these features were used as controls and actually decreasing in 2 of the 6 data sets, underscoring the need for caution and expert knowledge in the choice of control features like housekeeping genes.

These additional analyses are described in the main text in the “Mitigation strategies” section starting at line 320 and more thoroughly in the Supplement.

Minor comments:

Line 81 "residual variation in observed total abundances across samples is generally taken to be technical noise" - I would consider to refer to this as a technical bias, instead of "noise" which often connotes variance.

This is a good point. The wording has been updated.

Line 121 "low complexity" - what does this refer to specifically?
This was used as a synonym for low feature number but we admit the language is vague and potentially misleading. The verbiage has been changed in the main text.

Consideration - correlation of sequencing depth (total count) and the condition variable has been described as a pathological situation for differential abundance, in particular given that many null features may have zeros for the lower sequenced group. Was this explored in any of the simulations?
Simulated sequencing depth varied considerably across experiments but not very much within each experiment: total observed counts were similar within and across conditions for a given simulated data set. We agree this is an interesting topic for further exploration, especially at the extreme where “dropouts” occur as a direct result of reduced sampling in one condition, but suggest this additional analysis might be better suited to a broader study of batch effects in differential abundance testing.

Line 364 "txhese"
This has been fixed.

Line 391 - DESeq2 rescales using a simple method, just the median (over genes) of the ratio of each sample's count to the geometric mean over all samples. No Bayesian formula are used in this step.
This mischaracterization has been fixed in the text.

What versions of software were used?
All software versions have been added to the Methods section but are included here for clarity:

ALDEx2 version 1.22.0

ANCOM-BC version 1.0.5

DESeq2 version 1.30.1

edgeR version 3.32.1

scran version 1.18.5
